# IL-2 Therapy Diminishes Renal Inflammation and the Activity of Kidney-Infiltrating CD4+ T Cells in Murine Lupus Nephritis

**DOI:** 10.3390/cells8101234

**Published:** 2019-10-11

**Authors:** Angelika Rose, Caroline von Spee-Mayer, Lutz Kloke, Kaiyin Wu, Anja Kühl, Philipp Enghard, Gerd-Rüdiger Burmester, Gabriela Riemekasten, Jens Y. Humrich

**Affiliations:** 1 Charité – Universitätsmedizin Berlin, corporate member of Freie Universität Berlin, Humboldt-Universität zu Berlin, and Berlin Institute of Health, Department of Rheumatology and Clinical Immunology, Charitéplatz 1, 10117 Berlin, Germany; 2 German Rheumatism Research Center (DRFZ), a Leibniz Institute, Charitéplatz 1, 10117 Berlin, Germany; 3 Institute of Biotechnology, Department of Medical Biotechnology, Technical University Berlin, Gustav-Meyer-Allee 25, 13355 Berlin, Germany; 4 Charité – Universitätsmedizin Berlin, corporate member of Freie Universität Berlin, Humboldt-Universität zu Berlin, and Berlin Institute of Health, Department of Pathology, Charitéplatz 1, 10117 Berlin, Germany; 5 Charité – Universitätsmedizin Berlin, corporate member of Freie Universität Berlin, Humboldt-Universität zu Berlin, and Berlin Institute of Health, Medical Department (Gastroenterology, Infectious Diseases and Rheumatology) / Research Center ImmunoSciences (RCIS), Hindenburgdamm 30, 12200 Berlin, Germany; 6 Charité – Universitätsmedizin Berlin, corporate member of Freie Universität Berlin, Humboldt-Universität zu Berlin, and Berlin Institute of Health, Department of Nephrology and Intensive Care Medicine, Augustenburger Platz, 13353 Berlin, Germany; 7 University Hospital Schleswig-Holstein, Campus Lübeck, Department of Rheumatology and Clinical Immunology, Ratzeburger Allee 160, 23538 Lübeck, Germany

**Keywords:** SLE, lupus nephritis, regulatory T cell, interleukin-2, immunotherapy

## Abstract

An acquired deficiency of interleukin-2 (IL-2) and related disturbances in regulatory T cell (Treg) homeostasis play an important role in the pathogenesis of systemic lupus erythematosus (SLE). Low-dose IL-2 therapy was shown to restore Treg homeostasis in patients with active SLE and its clinical efficacy is currently evaluated in clinical trials. Lupus nephritis (LN), a challenging organ manifestation in SLE, is characterized by the infiltration of pathogenic CD4+ T cells into the inflamed kidney. However, the role of the Treg-IL-2 axis in the pathogenesis of LN and the mode of action of IL-2 therapy in the inflamed kidneys are still poorly understood. Using the (NZB × NZW) F1 mouse model of SLE we studied whether intrarenal Treg are affected by a shortage of IL-2 in comparison with lymphatic organs and whether and how intrarenal T cells and renal inflammation can be influenced by IL-2 therapy. We found that intrarenal Treg show phenotypic signs that are reminiscent of IL-2 deprivation in parallel to a progressive hyperactivity of intrarenal conventional CD4+ T cells (Tcon). Short-term IL-2 treatment of mice with active LN induced an expansion the intrarenal Treg population whereas long-term IL-2 treatment reduced the activity and proliferation of intrarenal Tcon, which was accompanied by a clinical and histological amelioration of LN. The association of these immune pathologies with IL-2 deficiency and their reversibility by IL-2 therapy provides important rationales for an IL-2-based immunotherapy of LN.

## 1. Introduction

Systemic lupus erythematosus (SLE) is a severe systemic autoimmune disease characterized by the breach of self-tolerance to nuclear autoantigens leading to inflammation and tissue destruction in multiple organ systems [1]. Lupus nephritis (LN) is a major contributor to the morbidity and mortality in SLE [2]. As early as 1984, Austin et al. linked renal inflammation with lymphocyte infiltration in LN and developed a scoring system for the histological assessment of LN [3]. Predominantly CD4+ T cells that belong to the Th1 lineage infiltrate the inflamed kidneys and are a predictor for the degree of renal inflammation in patients with LN [4,5,6,7]. Infiltrating CD4+ T cells thus play a crucial role for the initiation and perpetuation of inflammatory processes in LN. Studies that directly address the role of infiltrating immune cells in human organs are limited. Alternatively, the (NZB × NZW) F1 mouse model can be used as a suitable model to investigate the role of immune cells in the pathogenesis of LN [8].

Regulatory CD4+ T cells (Treg) that express the transcription factor FoxP3 are crucial for the control of autoimmunity by suppressing the activation and differentiation of self-reactive T cells and other pathogenic immune cells [9,10,11]. Their growth and survival fundamentally depend on the availability of the immunoregulatory cytokine interleukin-2 (IL-2) [12,13]. In the (NZB × NZW) F1 mouse model of lupus and in human SLE patients we found that an acquired deficiency of IL-2 caused a homeostatic disbalance between proliferating Treg and conventional CD4+ T cells (Tcon), which was accompanied by the loss of CD25 expression on Treg and an accelerated Tcon hyperactivity in lymphatic organs and peripheral blood [14,15]. These disturbances in the Treg-IL-2 axis were associated with increased disease activity, thereby highlighting the importance of IL-2 deficiency in SLE pathogenesis. Accordingly exogenous supplementation of IL-2 temporarily restored CD25 expression in Treg and the homeostatic balance between Treg and Tcon, and was capable to reduce disease activity in lupus-prone mice and also in SLE patients with active disease [14,15,16,17,18].

The coincidence of homeostatic and phenotypic abnormalities of the Treg population due to IL-2 deficiency with the development of LN proposes that an impairment of intrarenal Treg also contributes to renal inflammation. On the other hand, the fact that adoptive Treg transfers can delay the progression of LN [14,19] suggests that Treg may be directly involved in the regulation of pathogenic cells that infiltrate the inflamed kidneys. However, it is still unknown whether local or systemic IL-2 deficiency affects tissue-resident intrarenal Treg in analogy to lymphatic organs. In addition, the effects of IL-2 therapy on the composition and phenotype of intrarenal Treg and Tcon and the reversibility of organ-related pathologies associated with IL-2 deficiency are not well explored.

## 2. Materials and Methods

### 2.1. Mice

Female (NZB × NZW) F1 mice were bred at the Animal Facility of the Charité—University Medicine Berlin and kept under a special pathogen-free condition at the Deutsches Rheuma-Forschungszentrum (DRFZ), Berlin, Germany. All mice were grouped by age and disease activity and treated with recombinant mouse IL-2 (R&D Systems, Minneapolis, MN, USA) or sterile PBS according to institutional and federal guidelines (Landesamt für Gesundheit und Soziales, LaGeSo, Berlin, Germany).

### 2.2. Monitoring of Disease Activity and Definition of Disease Stages

Proteinuria was determined with Multistix 10 Visual (Siemens Healthcare Diagnostics Inc., Tarrytown, NY, USA). The following scoring system was used: Score 0 = 0 to 15 mg/dL, score 1 = 30 mg/dL, score 2 = 100 mg/dL, score 3 = 100–300 mg/dL, score 4 = 300 mg/dL and score 6 = 2000 mg/dL. The onset disease stage was defined as having a proteinuria score ranging from 0 to 3 and the nephritic stage as having a proteinuria score of >3.

### 2.3. Cell Preparation

For the analysis of peripheral blood derived cells, 50–200 µL of whole blood was taken from the tail veins of each animal at defined time points and before organs were harvested. Blood clotting was prevented by using heparin (Liquemin, Roche Pharma AG, Grenzach-Wyhlen, Germany) containing vials. Erythrocytes were lysed using a standard lysis buffer. Animals were sacrificed and spleens, lymph nodes (superficial inguinal and axillary) and one kidney were separately mashed through cell strainers (mesh size: 70 µm; BD Bioscience, Heidelberg, Germany) to obtain single cell suspensions. Suspensions were dissolved in PBS containing 0.2% BSA and 2 mM EDTA and were stained with fluorescently labeled antibodies. 

### 2.4. Flow Cytometry

Cells were stained with indicated antibodies in PBS containing 0.2% BSA. For extracellular staining the following fluorescently labeled antibodies were used: Anti-CD4-PerCP (GK1.5, eBioscience, Thermo Fisher Scientific, Waltham, MA, USA), anti-CD44-Pacific Blue (IM7, DRFZ), anti-CD69-FITC (H1.2F3, eBioscience) and anti-CD25-APC (PC61.5, eBioscience). For intracellular staining cells were fixed with fixation buffer and afterwards permeabilized with perm buffer (Foxp3/transcription factor staining buffer set, eBioscience) and then stained with anti-FoxP3-PE (FJK16s, eBioscience), anti-Helios-APC (22F6, Biolegend, San Diego, CA, USA) and anti-Ki67-Pe-Cy7 (B56, BD Bioscience) with the appropriate buffers according to the manufacturer’s protocol. Cells were stored in PBS containing 0.2% BSA and 0.01% sodium azide at 4 °C in the dark until measurement. Flow cytometry was performed using MACS Quant (Miltenyi Biotech, Bergisch Gladbach, Germany) and data were analyzed using FlowJo V 9.6.1 (BD Bioscience).

Gating strategy: 1. Lymphocyte gate in the FSC/SSC plot. 2. Gating either for CD4+FoxP3+ Treg or CD4+FoxP3− Tcon in a CD4/FoxP3 dot plot. 3. Frequencies of CD25+, CD69+, CD44hi, Ki67+ and Helios+ cells among gated CD4+FoxP3+ or CD4+FoxP3− cells were determined either by using different dot plot combinations or by using histograms.

### 2.5. Intracellular Cytokine Staining 

1 × 10^6^ total splenocytes were stimulated for 5 h with 20 ng/mL PMA and 1 µg/mL ionomycin (Sigma-Aldrich Chemie GmbH, Taufkirchen, Germany) together with Brefeldin A (Sigma-Aldrich) at a final concentration of 2 µg/mL. After stimulation, cells were stained with anti-CD4-Pe-Cy7 (GK 1.5, Biolegend) and anti-CD3-APCeFluor780 (17A2, eBioscience) in PBS and were afterwards fixed with BD Bioscience Cytokines Fixation Kit (according to manufacturer’s protocol). After fixation, cells were resuspended in 1× Perm buffer (BD Bioscience) and stained intracellularly with anti-IFNγ-FITC (XMG1.2, BD Bioscience), anti-IL-2-APC (JES6-5H4, Biolegend) and anti-CD44-Pacific Blue (IM7, DRFZ). 

### 2.6. Histological Analyses

Kidneys were fixed for at least 24 h in 4% paraformaldehyde immediately after harvesting. Afterwards, organs were dehydrated, embedded in paraffin and cut into 1–2 µm consecutive sections. Histochemical staining using hematoxylin and eosin (HE) as well as periodic acid Schiff (PAS) were performed. Sections were scored in a blinded manner by one independent pathologist using the renal activity index by Austin [3]. In detail, the activity index (AI) is the sum of six differentially weighted histomorphological scores, which are glomerular cell proliferation (×1), leukocyte exudation (×1), karyorrhexis and/or fibrinoid necrosis (×2), cellular crescents (×2), hyaline deposits (×2) and interstitial inflammation and tubulitis (×1). Each score was scaled from 0 to 3 (0 = no changes, 1 = mild changes, 2 = moderate changes and 3 = severe changes).

### 2.7. IL-2 Treatments

Disease activity and age-matched mice were injected subcutaneously either with 25 ng (2500 units) per gram bodyweight (between 500 ng and 1000 ng per injection) of recombinant mouse IL-2 (R&D systems, 402 ML) or with PBS alone every 24 h within the first five days (five times in total, short-term treatment). In the long-term treatments, IL-2 injections were continued every four days as maintenance treatment until day 29 (six additional injections). Mice were sacrificed and cells from different organs and peripheral blood were analyzed 12 h (short-term treatment) or 48 h (long-term treatment) after the last IL-2 injection. 

### 2.8. Statistical Analyses

Graph Pad Prism 5 software (GraphPad Software Inc., La Jolla, CA, USA) was used for the analysis. Statistical differences between two groups were analyzed by using the non-parametric Mann Whitney *U* test. Correlation analyses were performed by using Spearman’s rank correlation coefficients. Outliers were identified using the robust regression and outlier removal- (ROUT-) test. Cleared data were used for further statistical analyses and graphs. Differences were considered significant if *p* values were less than 0.05.

## 3. Results

### 3.1. Progressive Homeostatic Imbalance between Intrarenal Treg and Tcon

To obtain insights into the importance of Treg in the course of LN, we analyzed longitudinal changes in the numbers, frequencies and phenotype of intrarenal CD4+FoxP3+ Treg and of intrarenal CD4+FoxP3− Tcon during the progression of LN. 

**Figure 1:**
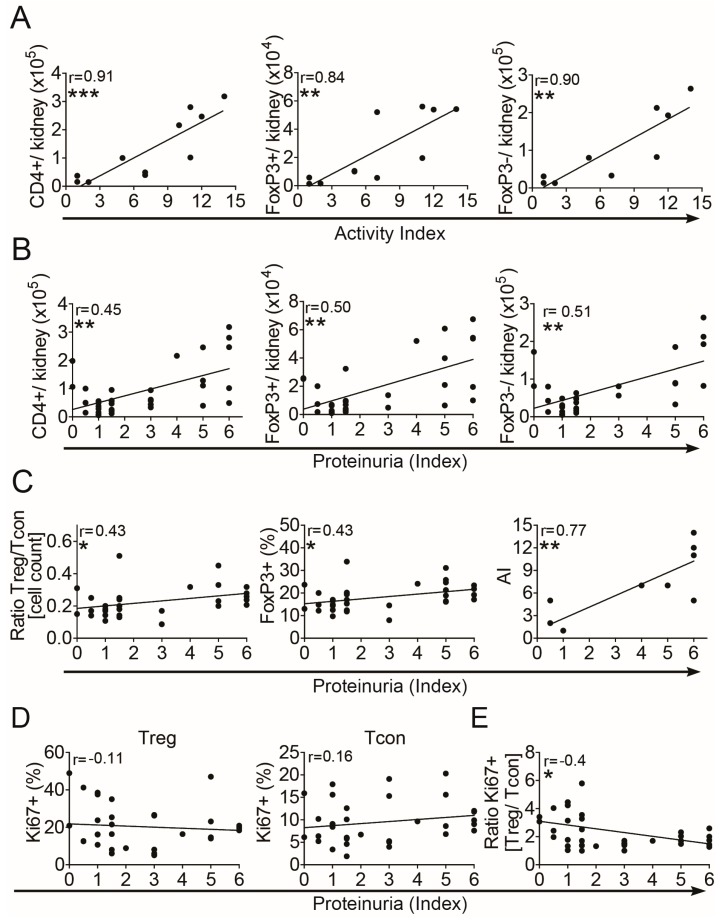
Progressive Treg/Tcon imbalance during progression of lupus nephritis. Cells from kidneys of (NZB × NZW) F1 mice at different disease stages were analyzed by flow cytometry. (**A**,**B**) Absolute numbers of total intrarenal CD4+ T cells, of intrarenal CD4+FoxP3+ Treg and of intrarenal CD4+FoxP3− Tcon in correlation with the renal histomorphological activity index (AI) (**A**) and with the proteinuria index (**B**). (**C**) Graphs show the correlation of the calculated ratio between absolute numbers of CD4+FoxP3+ Treg and CD4+FoxP3− Tcon (left graph), the correlation of the percentages of FoxP3+ cells among CD4+ T cells (middle graph) and the correlation of the AI with the proteinuria index (right graph). (**D**) The percentage of intrarenal Ki67+ cells among CD4+FoxP3+ Treg (left graph) and among CD4+FoxP3− Tcon (right graph) is shown. (**E**) The calculated ratio between percentages of Ki67+ Treg and of Ki67+ Tcon in correlation with the proteinuria index is shown. Data are derived from one kidney of each mouse from two to five independent experiments (*n* = 10 for correlations with AI; *n* = 28–30 for correlations with proteinuria index). Correlation analyses were performed by using Spearman’s rank correlation coefficients. Black lines indicate linear regression curves using Pearson analyses (* *p* < 0.05, ** *p* < 0.01 and *** *p* < 0.001).

First, we correlated the quantities of intrarenal Treg and Tcon with the corresponding histomorphological activity index (AI) and with the proteinuria index (PUI) in (NZB × NZW) F1 lupus prone mice with different renal activity. Increases in absolute numbers of total intrarenal CD4+ T cells, of intrarenal CD4+FoxP3+ Treg and of intrarenal CD4+FoxP3− Tcon significantly correlated with the AI and also with the PUI (Figure 1A,B). Calculation of the ratio between the absolute numbers of Treg and of Tcon revealed a more pronounced increase in Treg numbers than in Tcon numbers in correlation with the PUI (Figure 1C, left). Accordingly, the frequencies of intrarenal FoxP3+ Treg among CD4+ T cells also correlated with the PUI (Figure 1C, middle). In general, the PUI significantly correlated with the histomorphological AI (Figure 1C, right), suggesting that the PUI could be used as an alternative measure for the degree of renal inflammation.

Next we determined the frequencies of proliferating cells among intrarenal Treg and Tcon by using the proliferation marker Ki67. Here we could not observe significant correlations between the PUI and the frequencies of Ki67+ cells neither among Treg nor among Tcon, although a moderate decrease in Ki67+ Treg and a moderate increase in Ki67+ Tcon was apparent (Figure 1D). However, the calculated ratio between proliferating intrarenal Ki67+ Treg and Ki67+ Tcon, representing a measure of the homeostatic Treg/Tcon balance, continuously decreased in correlation with the PUI (Figure 1E). This suggests that a progressive homeostatic imbalance between Treg and Tcon also develops in the inflamed kidneys, comparable to findings in lymphatic organs and peripheral blood of these mice (Figure S1A) [14] and to findings in patients with SLE [15].

### 3.2. Phenotypic Changes of Intrarenal Treg

**Figure 2:**
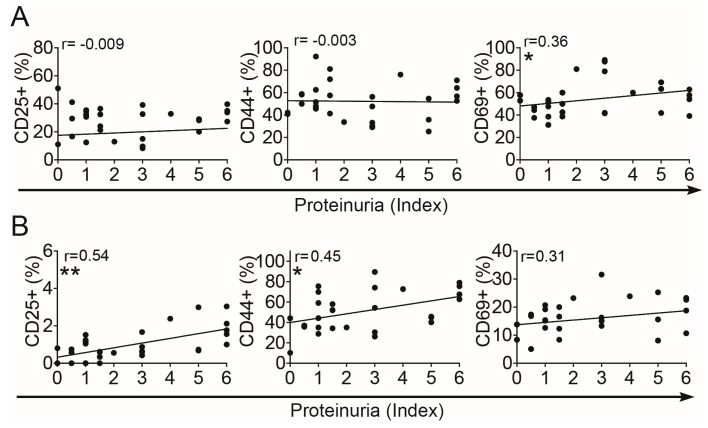
Phenotypic changes of intrarenal Treg and Tcon. Graphs show the percentage of CD25+, CD44hi and CD69+ cells among CD4+FoxP3+ Treg (**A**) and among CD4+FoxP3– Tcon (**B**) in correlation with the proteinuria index. Data are derived from one kidney of each mouse from two to five independent experiments (*n* = 26). Correlation analyses were performed by using Spearman’s rank correlation coefficients. Black lines indicate linear regression curves using Pearson analyses (* *p* < 0.05 and ** *p* < 0.01).

Next, we assessed whether intrarenal Treg develops typical phenotypic alterations that are associated with IL-2 deficiency during progression of LN, such as reduced expression of the IL-2 receptor α-chain, i.e., CD25, and increased expression of CD44 and CD69 as previously observed in the lymphatic organs and peripheral blood of these mice [14]. Therefore we correlated the frequencies of CD25, CD44 and CD69 expressing cells among Treg with the easy accessible PUI as an indicator of nephritic activity. The frequency of CD25+ cells among intrarenal Treg remained unchanged during progression of nephritis from the onset stage to the more active stages (Figure 2A). However, at the onset stage the frequency of CD25+ cells among intrarenal Treg was significantly lower compared to Treg from the spleens of the same disease stage and was in a similar range than in peripheral blood (Figure S1B, left). Of note, Treg from spleens and peripheral blood of (NZB × NZW) F1 mice at the onset stage of LN were shown to have already a reduced expression of CD25 when compared to Treg from age-matched BALB/c mice or to young (NZB × NZW) F1 mice [14]. Similar to observations in the spleens of (NZB × NZW) F1 mice, 50%–60% of the intrarenal Treg expressed the memory T cell marker CD44 [20] (Figure S1B, middle), which however did not considerably change in the kidneys during disease progression (Figure 2A, middle). CD69 has been considered to be a marker for recently activated T cells [21], however more recently it has been shown that CD69 also plays an important role in the retention of T cells in the inflamed organs [22]. The frequencies of CD69+ cells among intrarenal Treg significantly correlated with the PUI (Figure 2A, right) and were significantly higher than in the spleens at the disease onset (Figure S1B), suggesting a retention of intrarenal Treg at the site of inflammation and a high activity of Treg already in the early phase of LN, which further increases during the progression of LN. In conclusion, the phenotype of intrarenal Treg together with the progressive homeostatic Treg/Tcon imbalance suggests that a deficiency of IL-2 is also present in the inflamed kidneys of lupus-prone mice and such shortage of IL-2 occurs early during the course of LN.

### 3.3. Increased Activation of Intrarenal Tcon

In parallel to the Treg analyses, we assessed phenotypic changes of intrarenal Tcon during the progression of LN. The expression of CD25 is induced in Tcon upon activation independent of IL-2 availability and thus can be used as an activation marker for Tcon. The frequencies of CD25+ cells among intrarenal Tcon considerably correlated with the PUI as an indicator of nephritic activity (Figure 2B). The increases in the frequencies of CD25+ cells among Tcon between the onset stage and the nephritic stage were in a similar range than in the spleens and peripheral blood of these mice (Figure S1C). Corresponding to this, also the frequencies of CD44hi effector/memory cells among intrarenal Tcon markedly correlated with the PUI (Figure 2B). The frequencies of CD69+ cells among intrarenal Tcon were at the same level than in spleens (Figure S1C), but did not change considerably during progression of LN (Figure 2B). The increases in the frequencies of CD44hi cells among intrarenal Tcon between the onset and nephritic stage were similar to those in the spleens and peripheral blood (Figure S1C). These findings point to a progressive activation of intrarenal Tcon and an accumulation of effector/memory Tcon in the inflamed kidneys.

### 3.4. Decrease of Intrarenal IL-2 Producing CD4+ Memory/Effector T Cells in Active LN

**Figure 3:**
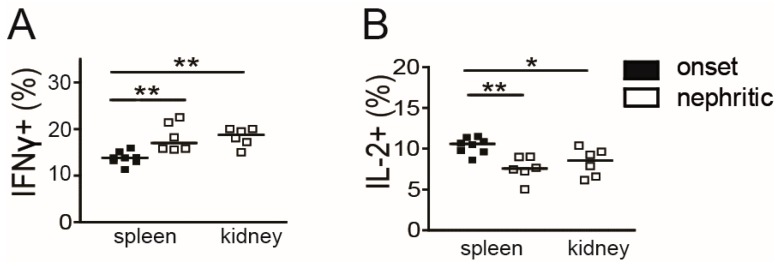
Low frequency of intrarenal IL-2 producing effector/memory T cells in active lupus nephritis. Sorted CD4+ T cells from single spleens and kidneys of individual mice at the indicated disease stage were stimulated with PMA/ionomycin for 5 h and the frequencies of IFN-γ (**A**) and IL-2 (**B**) producing cells among CD3+CD4+CD44hi memory T cells were determined by flow cytometry (onset: *n* = 8 and nephritic: *n* = 6). Horizontal lines represent the median. Mann-Whitney *U* test was used for statistical analyses (* *p* < 0.05 and ** *p* < 0.01).

To assess whether shortage of IL-2 is present in the inflamed kidneys at a cellular level as suggested by the data above, we determined the capability of intrarenal CD4+CD44hi memory/effector T cells to produce cytokines in comparison to splenic memory/effector T cells of (NZB × NZW) F1 mice. The frequency of IFN-γ producing cells among splenic CD4+CD44hi memory/effector T cells increased during progression from the onset to the nephritic stage (Figure 3A). In parallel and in line with previous findings in this lupus model [14], the frequency of IL-2 producing cells among splenic CD4+CD44hi T cells declined (Figure 3B). In nephritic mice, frequencies of IL-2 and of IFN-γ producing cells among intrarenal CD4+CD44hi T cells were in a similar range than in the spleens of nephritic mice (Figure 3A, B), suggesting that a decrease of IL-2 producing Tcon and a concomitant predominance of Th1 cells also occurs in the inflamed kidneys.

### 3.5. Short-Term IL-2 Treatment Expands the Intrarenal Treg Population

The data above suggested that IL-2 deficiency, either locally or systemically, might contribute to renal inflammation in (NZB × NZW) F1 mice. Therefore, (NZB × NZW) F1 mice with active nephritis were treated daily with a subcutaneous injection of recombinant IL-2 (25 ng/g body weight) for 5 consecutive days. Intrarenal CD4+FoxP3+ Treg and CD4+FoxP3− Tcon were analyzed 12 h after the last IL-2 injection and were compared to age- and disease activity-matched PBS-treated control mice. 

**Figure 4:**
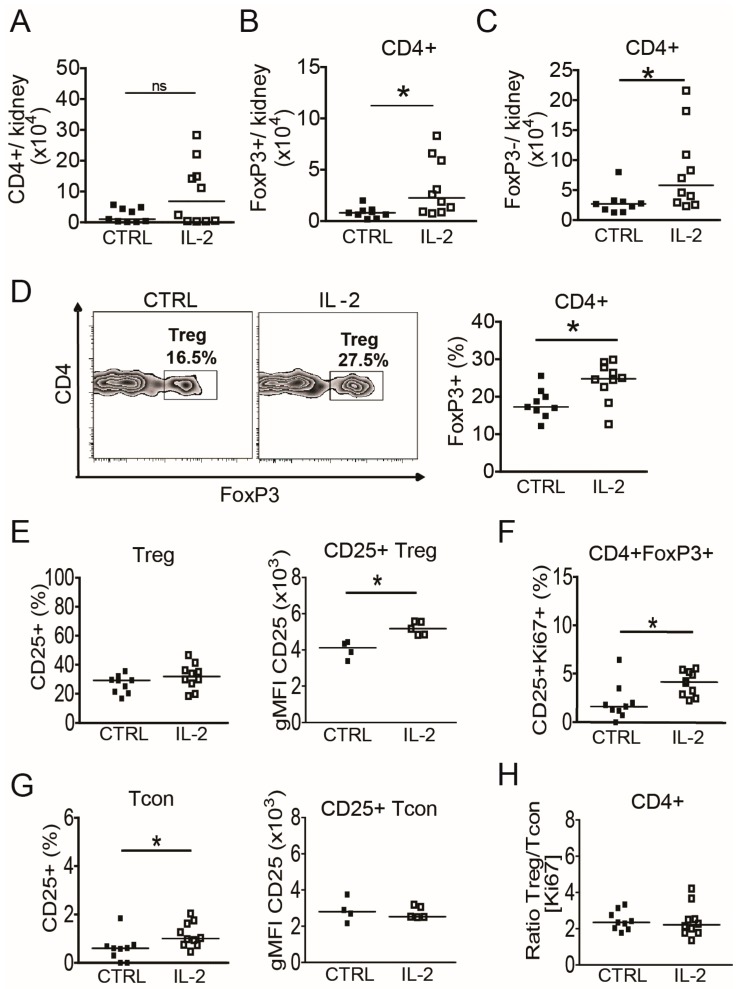
Short-term IL-2 treatment expands the intrarenal Treg population. Cells from kidneys of (NZB × NZW) F1 mice at the disease onset were analyzed by flow cytometry 24 h after a 5-day treatment course with daily injections of rIL-2 and compared with PBS-treated control mice. (**A**–**C**) Total numbers of intrarenal CD4+ T cells (**A**), of intrarenal CD4+FoxP3+ Treg (**B**) and of intrarenal CD4+FoxP3- Tcon (**C**) from IL-2 treated mice (IL-2) compared to control mice (CTRL) are shown. (**D**) Representative contour plots and scatter plot show the frequencies of FoxP3+ cells among CD4+ T cells in IL-2 treated mice compared to controls. (**E**) The frequency of CD25+ cells among CD4+FoxP3+ Treg and the geometric mean fluorescence intensity (gMFI) of CD25 in CD4+FoxP3+CD25+ Treg are shown. (**F**) The percentage of intrarenal CD25+Ki67+ cells among CD4+FoxP3+ Treg is shown. (**G**) The frequency of CD25+ cells among CD4+FoxP3− Tcon and the gMFI of CD25 in CD4+FoxP3-CD25+ Tcon are shown. (**H**) The calculated ratio between percentages of Ki67+ Treg and of Ki67+ Tcon is shown. Filled squares indicate PBS treated control mice (CTRL, *n* = 9) and open squares represent IL-2 treated mice (IL-2, *n* = 10). Horizontal lines represent the median. Mann-Whitney *U* test was used for statistical analyses (* *p* < 0.05).

Absolute numbers of total intrarenal CD4+ T cells, and more pronounced of CD4+FoxP3+ Treg and of CD4+FoxP3− Tcon increased approx. two-fold during the short-term IL-2 treatment compared to control mice (Figure 4A–C). The frequencies of FoxP3+ Treg among intrarenal CD4+ T cells were significantly higher in IL-2 treated mice compared to controls (Figure 4D), indicating that IL-2 preferentially expands the Treg population in the inflamed kidney. Although significant increases in the frequencies of CD25+ cells among Treg were observed in peripheral blood and spleens of IL-2 treated mice (Figure S3A), the frequency of CD25+ cells among intrarenal Treg was unaffected (Figure 4E, left). Nonetheless, the geometric mean fluorescence intensity (gMFI) of CD25 in intrarenal CD4+FoxP3+CD25+ Treg and of FoxP3 in intrarenal CD4+FoxP3+ Treg, representing the expression of CD25 and FoxP3 on a per-cell basis, was significantly higher compared to the control group (Figure 4E right; Figure S2). In addition, the frequencies of Ki67+CD25+ among intrarenal Treg were higher in the IL-2 treated mice compared to control mice (Figure 4F). This was also observed in peripheral blood but not in spleens of the same mice (Figure S3C), which suggests that systemically administered IL-2 has differing effects on Treg populations from different organs or compartments.

Analysis of intrarenal Tcon revealed higher frequencies of CD25+ cells among Tcon in short-term IL-2 treated mice compared to control mice (Figure 4G), which was not observed in spleens or peripheral blood (Figure S3B). However, and in contrast to intrarenal Treg, the gMFI of CD25 in intrarenal CD25+ Tcon remained unaffected (Figure 4G) and neither changes in the frequencies of intrarenal Tcon expressing Ki67, CD69 or CD44 (data not shown) nor changes in the Treg/Tcon proliferation ratio (Figure 4H) were observed in short-term IL-2 treated mice. Of note, the proliferation ratio of Treg/Tcon was significantly higher in the spleens of IL-2 treated mice, mainly due to a lower frequency of proliferating cells among Tcon (Figure S3D). Taken together, intrarenal Treg of lupus prone mice could be influenced by a short-term IL-2 treatment by means of numbers, phenotype and proliferation, whereas with the exception of an increase in the frequency of CD25+ Tcon, intrarenal Tcon were almost unaffected.

### 3.6. Long-Term IL-2 Treatment Diminishes the Activity of Intrarenal Tcon

**Figure 5:**
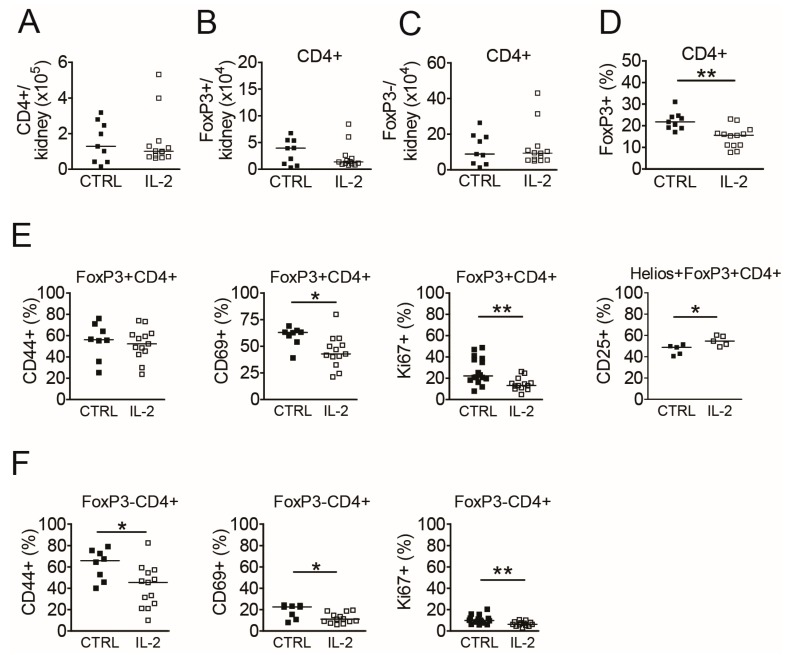
Long-term IL-2 treatment diminishes the activity and proliferation of intrarenal Treg and Tcon. Cells from kidneys of (NZB × NZW) F1 mice with active nephritis were analyzed by flow cytometry at day 31 after the initiation of the IL-2 treatments (48 h after the last IL-2 injection; IL-2, white bars) and were compared to PBS-treated control mice (CTRL, black bars). (**A**–**D**) Scatter plots show the absolute numbers of total CD4+ T cells (**A**), of CD4+FoxP3+ Treg (**B**) and of CD4+FoxP3− Tcon (**C**) and the percentage of FoxP3+ cells among CD4+ T cells in kidneys (**D**). (**E**–**F**) The percentages of CD25+, CD44hi, CD69+ and of Ki67+ cells among intrarenal CD4+FoxP3+ Treg (**E**) and among intrarenal CD4+FoxP3− Tcon (**F**) are shown. Filled squares indicate PBS treated control mice (CTRL; *n* = 8) and open squares represent IL-2 treated mice (IL-2, *n* = 13). Horizontal lines indicate the median of each group. Data are the summary of two to five independent experiments. Mann-Whitney *U* test was used for statistical analyses (* *p* < 0.05 and ** *p* < 0.01).

In addition to the short-term experiments, we next assessed the effects of long-term IL-2 treatments on intrarenal Treg and Tcon in comparison to spleens and peripheral blood from (NZB × NZW) F1 mice with active LN and whether there are differences in the AI determined by histomorphological analyses of the kidneys. Mice were treated with IL-2 on 5 consecutive days (five injections in total) followed by a maintenance treatment with repetitive IL-2 injections every four days for the duration of 29 days (six additional injections). Quantification of intrarenal CD4+ T cells at day 31 after the start of the IL-2 treatments (48 h after the last IL-2 injection) revealed no significant differences in the absolute numbers of total CD4+ T cells, of CD4+FoxP3+ Treg or of CD4+FoxP3- Tcon between IL-2 treated and control mice (Figure 5A–C). Unexpectedly, the frequency of intrarenal Treg among CD4+ T cells was significantly lower in the IL-2-treated mice compared to control mice (Figure 5D), which was not observed in spleens or in peripheral blood of these mice (Figure S4A).

Phenotypical analyses of intrarenal Treg showed lower frequencies of CD69+ and Ki67+ cells among Treg in IL-2 treated mice, whereas frequencies of CD44hi cells among Treg were unaffected by the long-term IL-2 treatments (Figure 5E). Comparable changes in the frequencies of CD69+ and Ki67+ Treg could be observed in the spleens but not in the peripheral blood of IL-2 treated mice (Figure S4D). Although frequencies of CD25+ cells among total intrarenal Treg did not increase in IL-2 treated mice (data not shown), which was different to findings in the spleens (Figure S4D), we found significantly higher frequencies of CD25+ cells among the Helios+FoxP3+ Treg subset, which is considered to be of thymic origin [23], in the kidneys and also in spleens of IL-2 treated mice (Figure 5E, Figure S5).

**Figure 6:**
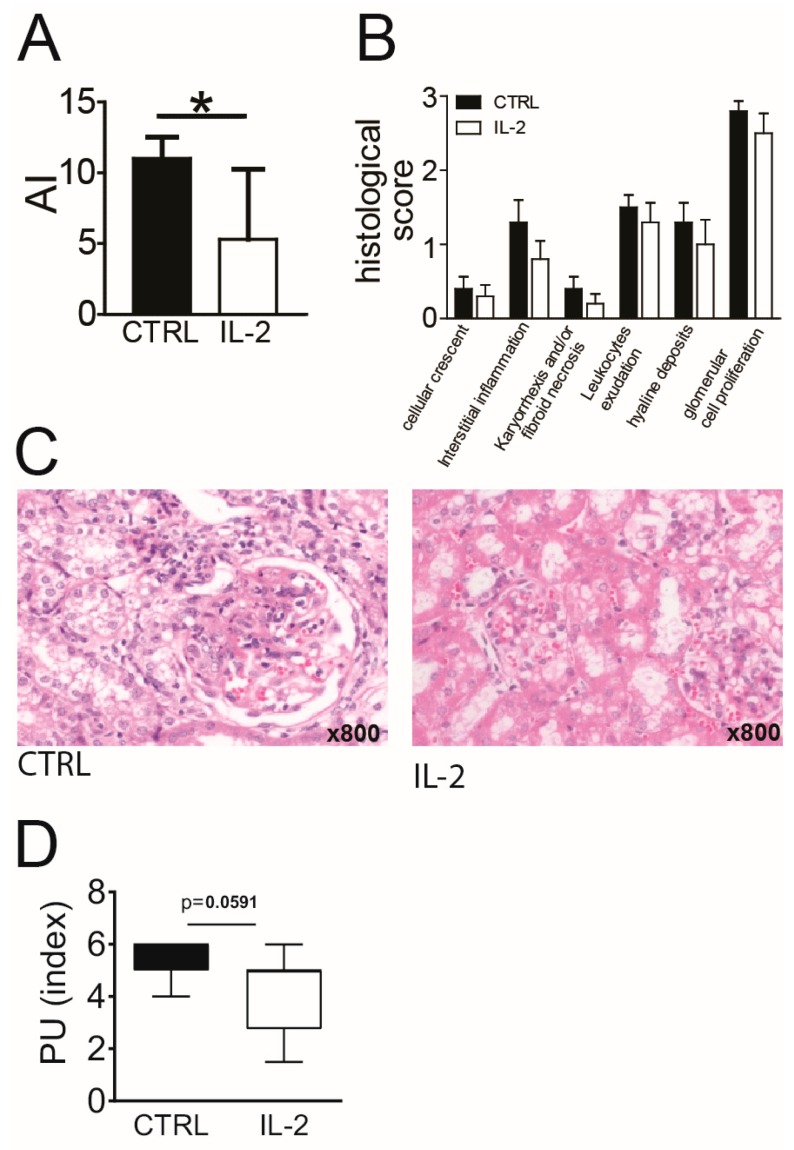
Long-term IL-2 treatment reduces nephritic activity at a histological level. (**A**) The renal activity index (AI) was determined in single kidneys from (NZB × NZW) F1 mice with established nephritis at day 31 after the initiation of the IL-2 treatments (48 h after the last IL-2 injection; IL-2, white bars) and compared to PBS-treated control mice (CTRL, black bars). (**B**) The six differentially weighted histomorphological scores according to the AI are shown for the control and the IL-2 treated group. (**C**) Representative images of hematoxylin and eosin stained kidney sections from either controls (left) or from IL-2 treated mice (right) are shown. (**D**) The proteinuria index of controls and IL-2 treated mice at day 31 are shown. Data represent the mean + SD of the scores summarized from two to five independent experiments (*n* = 6–17). Mann-Whitney U test was used for statistical analyses (* *p* < 0.05 and ** *p* < 0.01). One outlier in the CTRL group at d0 was identified and removed after using the ROUT test.

In parallel to the effects on intrarenal Treg, we observed significantly lower frequencies of CD44hi effector/memory cells among intrarenal Tcon in IL-2 treated mice compared to controls (Figure 5F). In addition, frequencies of CD69+ cells and of proliferating, Ki67+ cells among intrarenal Tcon were significantly lower in IL-2 treated mice compared to the control group (Figure 5F). Frequencies of CD25+ cells among intrarenal Tcon did not change significantly under the IL-2 treatment (not shown). Of note, frequencies and phenotype of intrarenal Treg and Tcon in long-term IL-2 treated mice with active LN were in a comparable range as in (NZB × NZW) F1 mice at the onset stage (Figure 1). Similar, but less pronounced, changes in the frequencies of CD25+, CD44hi, CD69+ and Ki67+ Tcon were observed in spleens, but not in the peripheral blood of long-term IL-2 treated mice (Figure S4E). Simultaneously performed histological analyses of the kidneys showed a significantly lower AI in IL-2 treated mice compared to control mice (Figure 6A–C). Complementary to this IL-2 treated mice also had a lower PUI than control mice at day 31 (Figure 6D).

## 4. Discussion

An acquired deficiency of IL-2 profoundly affects Treg biology in lupus-prone individuals and significantly contributes to the immune pathogenesis of SLE [14,24]. Low-dose IL-2 therapy currently receives major attention in medicine as a novel and unique therapeutic approach, which is different from conventional treatment strategies that commonly induce global immunosuppression [25,26]. Low-dose IL-2 therapy intends to restore Treg homeostasis and early phase clinical trials suggest that this treatment is efficacious in large variety of autoimmune diseases including SLE [17,18,25,27,28]. Although it is undisputable that low-dose IL-2 therapy is capable to expand the Treg population very efficiently and also quite selectively in virtually every treated patient, the mechanisms of action of low-dose IL-2 therapy beyond Treg expansion are still not well understood [18,28,29]. In particular, deeper insights into the IL-2 induced immunological changes in the inflamed tissues are necessary for a better understanding of the modes of action of low-dose IL-2 therapy. In this study, we addressed whether kidney-infiltrating Treg are affected by a shortage of IL-2 and whether immune pathologies in the inflamed kidneys can be influenced by exogenous administration of IL-2.

We found that already at the onset stage of LN, intrarenal Treg showed a reduced expression of CD25, which was even lower than in Treg from spleens of these mice. The low expression of CD25 on Treg represents a hallmark of IL-2 deficiency [13,14] and thus suggests an insufficient availability of intrarenal IL-2 already at the early phase of LN. Complementary to these abnormalities we found low frequencies of IL-2 producing cells among intrarenal effector/memory Tcon in mice with active nephritis. The reduced availability of IL-2 in the inflamed kidneys of (NZB × NZW) F1 lupus-prone mice could therefore result in impaired Treg growth and survival thereby impeding their capability to adequately control the progressive hyperactivity of intrarenal effector/memory Tcon. Based on these findings, it would be plausible to assume that numbers and frequencies of intrarenal Treg decline during the progression of LN. Instead we observed an increase in both, the numbers and the frequencies of Treg during the course of LN indicating a continuous enrichment or expansion of the Treg population in the inflamed kidneys. Similar observations were made for Treg in the spleens of these mice and are also reported for human SLE patients, where the frequencies of CD4+FoxP3+ Treg in the peripheral blood correlated with disease activity [15,30]. In addition, already at the onset stage a large proportion of intrarenal Treg expressed CD69 (a marker for tissue-resident cells and T cell activation) suggesting that intrarenal Treg are in a highly active state and are retained in the inflamed kidney. Overall these observations suggest a counter-regulatory mechanism attempting to dampen cellular hyperactivity in LN that however may fail due to an insufficient availability of growth factors, such as IL-2 [14,24].

Another hallmark of IL-2 deficiency in SLE is the development of an imbalanced proliferation between Treg and Tcon in favor of Tcon in lymphatic tissues and the peripheral blood [14,15]. Although the proportions of proliferating cells among intrarenal Treg only moderately declined in parallel to a moderate increase in the proportions of proliferating Tcon, the calculated Treg/Tcon proliferation ratio, as a measure of the homeostatic balance between Treg and Tcon, significantly declined during the progression of LN. This is analogous to findings in lymphatic organs of these mice [14] and indicates that a progressive homeostatic Treg/Tcon imbalance develops also in the inflamed kidneys. This also suggests that intrarenal Treg have a reduced capacity to efficiently control the expansion of pro-inflammatory intrarenal Tcon. Thus, the progressive increase in frequencies and quantities of intrarenal Treg that we observed during the course of LN might be the result of enhanced tissue recruitment in order to compensate for the loss of control over Tcon activity in a numerical way [14,24]. Yet, the parallel increase in the numbers of kidney-infiltrating Tcon and their increased activation proposes that during progression of LN Treg lose control over Tcon activation in the inflamed kidneys.

Based on these findings we hypothesized that systemic administration of IL-2 also directly affects intrarenal Treg and thereby corrects the observed Treg defects in the kidneys. We found that short-term IL-2 treatment for five consecutive days was capable to increase the numbers and frequencies of intrarenal Treg. Simultaneously the expression levels of CD25 (gMFI) on Treg and the proportion of proliferating CD25+ Treg were significantly augmented in the kidneys. This indicates that intrarenal Treg can be targeted and modulated by IL-2 therapy, although the effects on intrarenal Treg appeared to be in part different to those observed in spleens and peripheral blood. The reasons for these organ-related differences in Treg responsiveness are unclear, but we suppose that this may be due to differences in the distribution and clearance of IL-2 in the organism. It is known that kidneys are the major site of clearance of circulating IL-2 in the body, which is mediated by both glomerular filtration and peritubular extraction [31]. Thus, it could be speculated that intrarenal Treg are exposed to higher levels of IL-2 than Treg in other organs. The transient increase in the frequencies of CD25+ cells among intrarenal Tcon during the short-term IL-2 treatment is in line with recent findings in peripheral blood of SLE patients treated with low doses of IL-2 and may indicate a certain lack of selectivity for Treg [18]. Yet, in consideration of the amelioration of LN and the reduction in activated intrarenal Tcon that was observed during the long-term IL-2 treatment, such a transient activation of intrarenal Tcon may be clinically rather negligible.

Long-term IL-2 treatment led to a decreased activity and proliferation of intrarenal Tcon and in parallel resulted in an amelioration of renal inflammation assessed by histomorphological scoring of the kidney. The fact that the reduction of Tcon hyperactivity in the kidneys was only observed in long-term treated mice and not in the short-term setting, suggests that multistep immunoregulatory processes, most likely mediated by Treg, took place in the kidneys, and that direct and rapid effects of IL-2 on the Tcon population, such as the induction of activated-induced cell death [32,33], were not predominant in this setting.

Unexpectedly, and in contrast to findings in peripheral blood and spleens and also different to the short-term treatment, the frequencies and numbers of intrarenal Treg and their proliferation and activation state was lower in long-term IL-2 treated mice compared to controls. At first view, such findings under long-term IL-2 treatment seem to be contradictory. However, we suppose that the reduced quantities and activation state of intrarenal Treg under long term IL-2 treatment are the consequence of the reduced activity of intrarenal Tcon, leading also to a lesser requirement of counteracting Treg in the kidneys. Of note, both, frequencies and absolute numbers of intrarenal Treg in long-term IL-2-treated mice were in the same range as in mice at the onset stage with less active LN. Overall these findings further support that long-term IL-2 treatment is capable to decelerate or reverse Tcon activity in the affected organs. Although no increase in the frequencies of CD25+ among total Treg was observable in the kidneys of long-term IL-2 treated mice we found a significantly higher frequency of CD25+ cells among the Helios+ Treg subset, suggesting a preferential targeting of thymic-derived Treg by IL-2 therapy.

The results of our study are complementary to recent findings in the MRL/lpr mouse model of SLE where it was shown that systemic administration of low doses of IL-2 decreases the numbers of IL-17- producing, so called, double negative T cells in the spleens and in parallel reduces inflammation in the kidneys and other affected organs [34]. More recently it was also shown in the (NZB × NZW) F1 model that treatment with IL-2/anti-IL-2 complexes induced an expansion of the Treg population in the kidneys, which was accompanied by a reduction of kidney-infiltrating IFN-γ+CD4+ and IL-17A+CD4+ T cells and an amelioration of LN at a histomorphological and clinical level [35]. These data together with our findings reasonably support the implementation of an IL-2-based immunotherapy in the treatment of LN.

Limitations of our study include that we were not able to assess the suppressive function of intrarenal Treg because of limited amounts of cells that can be isolated and sorted from the kidneys. In addition, more detailed analyses of the effects of IL-2 on intrarenal T cell subsets, including Th1, Th17, T follicular helper cells and CD8+ T cells or B cell subsets and NK cells are required for a deeper understanding of the complex immunological processes that occur in the inflamed tissues under IL-2 therapy. Moreover, the diversity of potential suppressive mechanisms of Treg that take place in the inflamed kidney including Treg-mediated cytotoxic effects on Tcon and other harmful immune cells is well worth to be addressed in future studies.

In summary, our study proposed that a disturbance of the Treg-IL-2 axis contributed to the pathophysiology of LN and particularly promoted the hyperactivity of intrarenal Tcon. The reversibility of these immune pathologies in affected organs by IL-2 therapy shown here provided valuable insights into the mechanisms of action of IL-2 therapy at a cellular level and offered additional rationales for an IL-2-based immunotherapy of LN.

## Supplementary Materials

The following are available online at https://www.mdpi.com/2073-4409/8/10/1234/s1, Figure S1: Phenotypic changes of Treg and Tcon in different organs during progression of LN, Figure S2: Short-term IL-2 treatment increases the expression of FoxP3 in intrarenal Treg, Figure S3: Phenotypic changes of Treg and Tcon in spleens and peripheral blood after short-term IL-2 treatment, Figure S4: Phenotypic changes of Treg and Tcon in spleens and peripheral blood after long-term IL-2 treatment, Figure S5: Long-term IL-2 treatment increases CD25 expression in Helios+ Treg in the spleen.

## Abbreviations

AI, activity index; gMFI, geometric mean fluorescence intensity; IL-2, interleukin 2; LN, lupus nephritis; NZB, New Zealand Black; NZW, New Zealand White; PUI, proteinuria index; SLE, Systemic Lupus erythematosus; Tcon, conventional T cells; Treg, regulatory T cells.
