# Supplementary material for: IL-2 Therapy Diminishes Renal Inflammation and the Activity of Kidney-Infiltrating CD4+ T Cells in Murine Lupus Nephritis
Source: Cells. 2019 Oct 11;8(10):1234. doi: 10.3390/cells8101234 (PMC6829607; doi:10.3390/cells8101234)
Supplement: Supplementary file 1 [file cells-08-01234-s001.pdf]

Supplementary Figures

Rose et al. **IL-2 therapy diminishes renal inflammation and the activity of kidney-infiltrating CD4+ T cells in murine lupus nephritis**

Figure S1

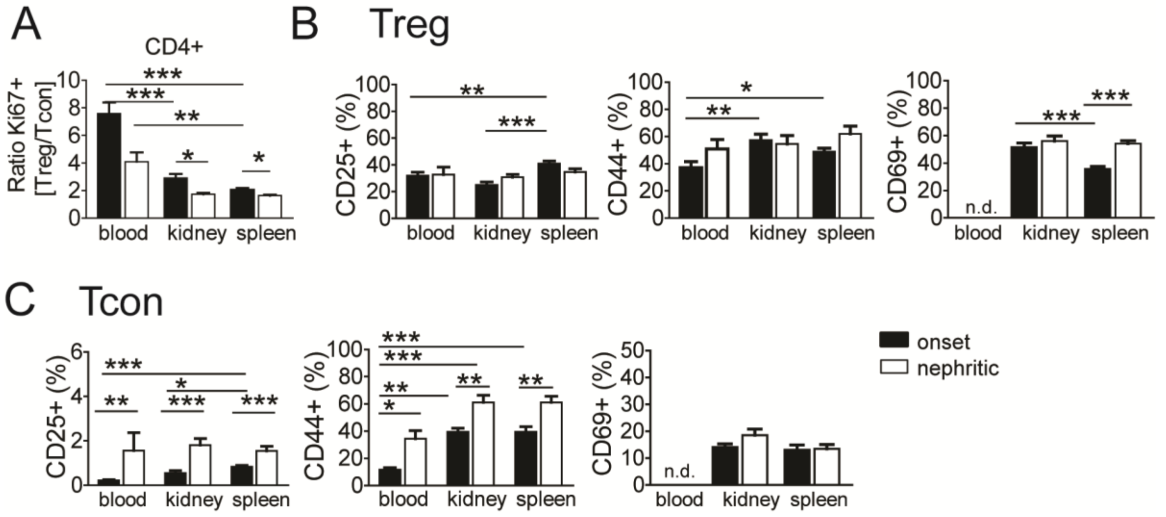

Figure S1. Phenotypic changes of Treg and Tcon in different organs during progression of LN. Cells from peripheral blood, kidneys and spleens of (NZB × NZW) F1 mice at the disease onset (onset) and with established nephritis (nephritic) were analyzed by flow cytometry. (**A**) The calculated ratio between percentages of Ki67+ Treg and of Ki67+ Tcon is shown. (**B,C**) The percentages of CD25+, CD44hi, CD69+ cells among CD4+FoxP3+ Treg (**B**) and among CD4+FoxP3- Tcon (**C**) is shown. Mice were grouped according to their proteinuria score into onset (PU Score ≤3) and nephritic (PU Score >3). Data are the summary of four to six independent experiments. Horizontal lines indicate the median + SEM of each group. Mann-Whitney U test was used for statistical analyses (*p≤0.05, **p<0.01, ***p<0.001).

Figure S2

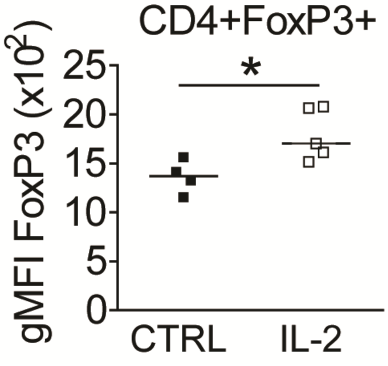

Figure S2. Short-term IL-2 treatment increases the expression of FoxP3 in intrarenal Treg. Cells from kidneys of (NZB × NZW) F1 mice with established disease were analyzed by flow cytometry 24 h after a 5-day treatment course with daily injections of rIL-2 and compared with PBS-treated control mice. The geometric mean fluorescence intensity (gMFI) of FoxP3 within intrarenal CD4+FoxP3+ cells is shown. Data are from one experiment. Filled squares indicate PBS treated control mice (CTRL, n=5) and open squares represent IL-2 treated mice (IL-2, n=4). Horizontal lines indicate the median of each group. Mann-Whitney U test was used for statistical analyses (*p<0.05).

Figure S3

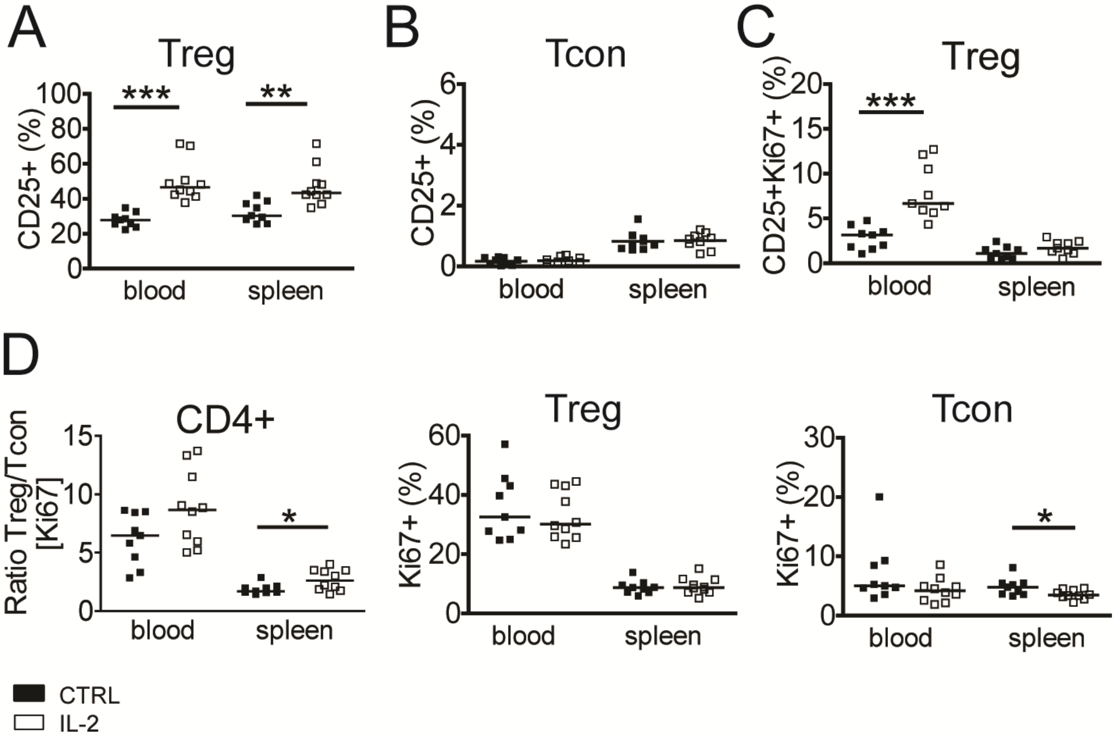

Figure S3. Phenotypic changes of Treg and Tcon in spleens and peripheral blood after short-term IL-2 treatment. Cells from spleens and peripheral blood of (NZB × NZW) F1 mice at the disease onset were analyzed by flow cytometry 24h after a 5-day treatment course with daily injections of rIL-2 and compared with PBS-treated control mice. (**A-C**) Frequencies of CD25+ cells among FoxP3+CD4+ Treg (**A**), of CD25+ cells among FoxP3-CD4+ Tcon (**B**) and of CD25+Ki67+ cells among FoxP3+CD4+ Treg (**C**) are shown. (**D**) The calculated ratio between percentages of Ki67+ Treg and Ki67+ Tcon and percentages of Ki67+ cells among Treg and Tcon are shown. Filled squares indicate PBS treated control mice (CTRL) and open squares represent IL-2 treated mice (IL-2). Data are the summary from two independent experiments. Horizontal lines indicate the median of each group. Mann-Whitney U test was used for statistical analyses (*$p \leq 0.05$, **$p<0.01$, ***$p<0.001$). One outlier in the CD25+Ki67+ Treg subset of the IL-2 group at day 5 (blood and spleen) was identified and removed after using the ROUT test.

Figure S4

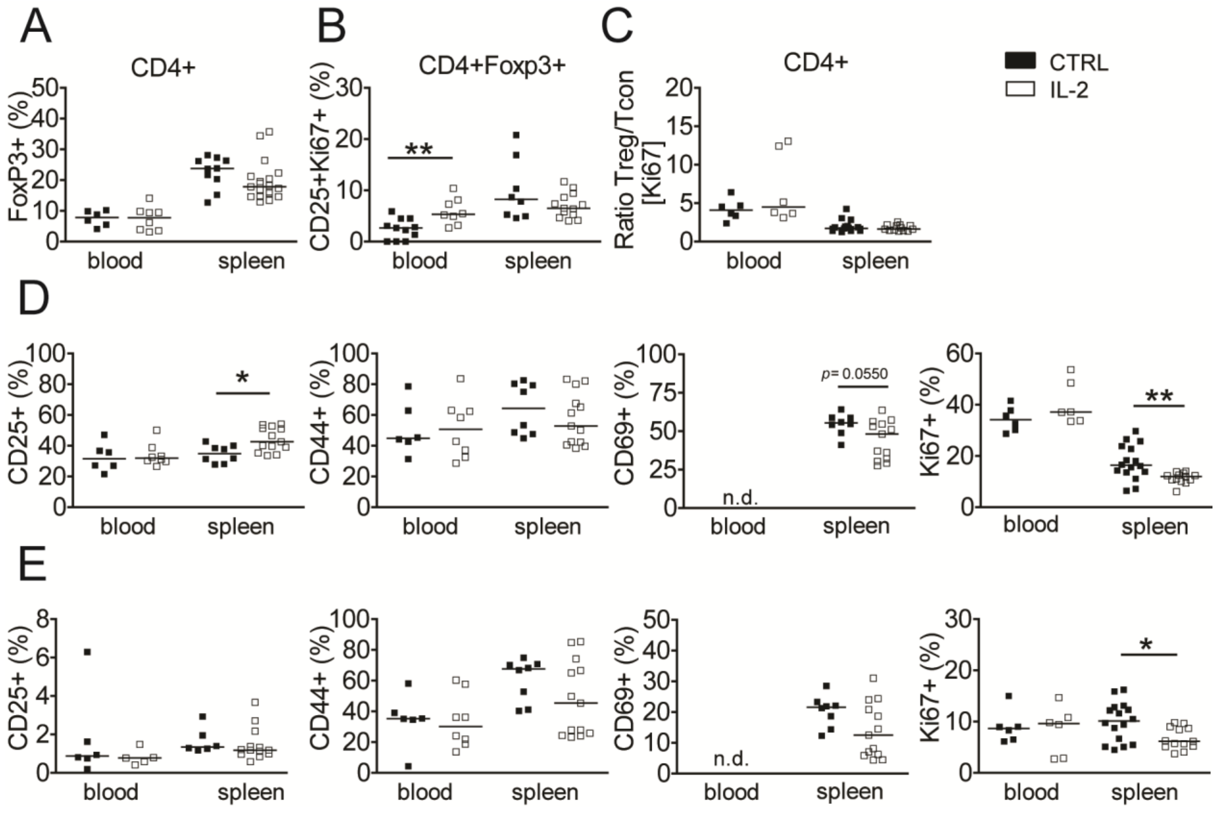

Figure S4. Phenotypic changes of Treg and Tcon in spleens and peripheral blood after long-term IL-2 treatment. Cells from spleens and peripheral blood of (NZB × NZW) F1 mice with active nephritis were analyzed by flow cytometry at day 31 after the start of the IL-2 treatment (48h after the last IL-2 injection) (IL-2, white bars) and were compared to PBS-treated control mice (CTRL, black bars). (**A-C**) Frequencies of FoxP3+ Treg among CD4+ T cells (**A**), of CD25+Ki67+ cells among CD4+FoxP3+ Treg (**B**) and the calculated ratio between percentages of Ki67+ Treg and Ki67+ Tcon are shown. (**D, E**) Frequencies of CD25+, of CD44hi, of CD69+ and of Ki67+ cells among CD4+FoxP3+ Treg (**D**) and among CD4+FoxP3- Tcon (**E**) are shown. Filled squares indicate PBS treated control mice (CTRL) and open squares represent IL-2 treated mice (IL-2). Data represent the summary from two independent experiments. Horizontal lines indicate the median of each group. Mann-Whitney U test was used for statistical analyses (*$p \leq 0.05$, **$p < 0.01$).

Figure S5

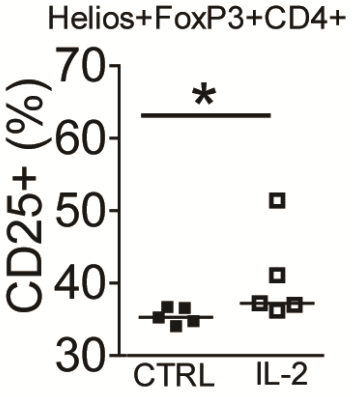

Figure S5. Long-term IL-2 treatment increases CD25 expression in Helios+ Treg in the spleen. The frequencies of CD25+ cells among splenic CD4+FoxP3+Helios+ Treg were analyzed by flow cytometry in nephritic (NZB × NZW) F1 mice at day 31 after the start of the IL-2 treatment. Filled squares indicate PBS treated control mice (CTRL, n=5) and open squares represent IL-2 treated mice (IL-2, n=5). Horizontal lines indicate the median of each group. Mann-Whitney U test was used for statistical analyses (*p<0.05).
